# Tetramethylpyrazine Protects against Early Brain Injury after Experimental Subarachnoid Hemorrhage by Affecting Mitochondrial-Dependent Caspase-3 Apoptotic Pathway

**DOI:** 10.1155/2017/3514914

**Published:** 2017-02-27

**Authors:** Shaoxue Li, Xiaolan Xiao, Xiaojia Ni, Zhennan Ye, Junjie Zhao, Chunhua Hang

**Affiliations:** ^1^Department of Neurosurgery, School of Medicine, Southern Medical University, Jinling Hospital, 305 East Zhongshan Road, Nanjing, Jiangsu Province 210002, China; ^2^Guangdong Provincial Hospital of Chinese Medicine, Guangdong Provincial Academy of Chinese Medical Sciences, The Second Clinical School, Guangzhou University of Chinese Medicine, Guangzhou, Guangdong Province 510120, China; ^3^Surgery & Interventional Science, Royal Free Hospital, University College London Medical School, Pond Street, London, UK

## Abstract

This study was to test the hypothesis that tetramethylpyrazine (TMP) protected against early brain injury after subarachnoid hemorrhage (SAH) by affecting the mitochondrial-dependent caspase-3 apoptotic pathway. TMP was administrated after the rats' prechiasmatic SAH mode. Animal neurobehavioral functions were assessed and the mitochondrial morphology, mitochondrial and cytoplasmic calcium, and mitochondrial membrane potential changes (Δ*ψ*m) of the brain tissues were measured. The expressions of cytoplasmic cytochrome c (cyt c), second mitochondria-derived activator of caspases (Smac), and cleaved caspase-3 B-cell lymphoma 2 (bcl-2) in cells were determined and cellular apoptosis was detected. The treatment of TMP resulted in less apoptotic cells and milder mitochondrial injury and potentially performed better in the neurobehavioral outcome compared to those with saline. Also, TMP ameliorated calcium overload in mitochondria and cytoplasm and alleviated the decrease of Δ*ψ*m. In addition, TMP inhibited the expression of cytoplasmic cyt c, Smac, and cleaved caspase-3, yet it upregulated the expression of bcl-2. These findings suggest that TMP exerts an antiapoptosis property in the SAH rat model and this is probably mediated by the caspase-3 apoptotic pathway triggered by mitochondrial calcium overload. The finding offers a new therapeutic candidate for early brain injury after SAH.

## 1. Introduction

Subarachnoid hemorrhage (SAH), accounting for 5% of all strokes, is a fatal disease with high morbidity and mortality, and most cases result from the rupture of intracranial aneurysm [1]. Early brain injury (EBI) is considered as the primary cause of mortality as well as the deciding factor to prognosis for survivors [2].

Many pathological processes are involved after the initial hemorrhage such as apoptosis, oxidative stress, and impaired calcium homeostasis [3]. Cell apoptosis plays a dominant role in developing EBI after SAH [4]. This process is probably triggered by the mitochondrial injury [5]. Overloading Ca^2+^ is commonly known as one of the indicators of mitochondrial injury [6]. It could initiate a molecular cascade that culminates in cell apoptosis [7], including promoting the depolarization of the mitochondria, then releasing second mitochondria-derived activator of caspases (Smac) and cytochrome c (cyt c) to the cytoplasm, enhancing caspase 3, and finally activating deoxyribonuclease (DNase) to degrade DNA of the neurons [6, 8]. At the same time, the release of cyt c can increase the production of B-cell lymphoma 2 (bcl-2), to inhibit the initiation of cell apoptosis [6, 9, 10]. Therefore, ameliorating mitochondrial injury could be a promising therapeutic target for early brain injury after SAH.

Chuanxiong rhizome (scientific name:* Ligusticum chuanxiong Hort*.; Chinese name:* chuanxiong*) has been widely used to treat cerebrovascular diseases with a long history in China [11]. Tetramethylpyrazine (TMP), an active ingredient extracted from chuanxiong rhizome, demonstrates a broad therapeutic capacity such as scavenging oxygen free radicals, reducing the migration of circulatory leukocytes, downregulating proinflammatory cytokine production, and regulating the NO/cGMP signaling [12–16]. In animal models with cerebral or spinal cord ischemia, TMP reduces cerebral infarct volumes, which is probably mediated by rescuing neuronal apoptosis [12, 13, 17–19]. This antiapoptotic effect is associated with downregulating the expressions of bcl-2 and Bcl-2 Associated X Protein (bax) alongside with suppressing caspase-3 [12].

In the animal model with SAH, TMP inhibits cell apoptosis as well, which is related to reducing caspase-3 in the cytoplasm [20]. However, it remains uncertain whether this effect has a relationship with rescuing mitochondrial dysfunction though TMP relieves mitochondrial impairments in other animal models [21]. Therefore, we hypothesized that the antiapoptotic effect of TMP was associated with altering the mitochondrial-dependent caspase-3 apoptotic pathway. To validate this hypothesis, we conducted the neurobehavioral assessment of the SAH rats treated by TMP and measured mitochondrial and cytoplasmic Ca^2+^ accumulation and mitochondrial membrane potential changes alongside with proteins expressed in the following caspase-3 apoptotic pathway.

## 2. Materials and Methods

All procedures were conducted in accordance with the UK Animals (Scientific Procedures) Act 1986 and associated guidelines, the EEC Directive of 1986 (86/609/EEC), the NIH guide for the care and use of laboratory animals (NIH Publication number 80-23; revised 1978), the Regulations for the Administration of Affairs concerning Experimental Animals released by the State Council of the People's Republic of China (number 2, 1988), and Guidelines for the Care and Use of Laboratory Animals published by Ministry of Science and Technology of the People's Republic of China (number 2, 1988).

### 2.1. Animal Preparation and Grouping

Adult male Wistar rats (300 g–330 g) were obtained from the Animal Center, Yangzhou University. All rats were housed in 12 h dark/light cycle room with the average temperature of 25°C, and they were allowed free access to food and water. Seventy-five rats were randomly allocated to three groups, including SAH model treated with TMP (*n* = 25), SAH model treated with saline (*n* = 25), and the sham-operation group without any treatments (*n* = 25).

### 2.2. SAH Model

As previously described [22], the SAH model was established by injecting autologous blood to prechiasmatic cistern of the rats. First, the Wistar rats were anesthetized with chloral hydrate (400 mg/kg, IP). Then 300 *μ*L blood was drawn from the right femoral artery with an insulin syringe (1 mL 29 G × 1/2 m, 0.33 mm × 12.7 mm) (BD Science, US). A midline scalp incision was made and a 1 mm hole was drilled 8.0 mm anterior to the bregma in the midline of the skull without brain injury. Subsequently, the needle of the syringe was advanced 11 mm at a 45° angle into the prechiasmatic cistern through the burr hole. The procedure in the sham-operation group was identical except saline was injected instead. The SAH model was confirmed by autopsy.

### 2.3. Treatments

TMP was obtained from Shanghai Yuanye Bio-Technology, Shanghai, China (number KM0513CA14). It was dissolved in saline water. TMP at 30 mg/kg was administrated by intraperitoneal injection 15 minutes after the experimental SAH because this treatment was proven to inhibit the caspase-3 [20] though effective doses of TMP for ischemia varied (10 mg/kg to 160 mg/kg) [12, 19, 23]. As a control group, 3 mL saline was injected to the SAH rats instead.

### 2.4. Neurobehavioral Assessment

Neurobehavioral assessment containing appetite, activity, and neurological deficits was conducted 24 hours after the experimental SAH [8]. Four grades of impairments were assigned to the rats according to the total scores, including no impairment (score = 0), slight impairment (scores ≤ 2), moderate impairment (scores = 3~4), and severe impairment (scores ≥ 5). The assessor was blind to the treatment allocations.

### 2.5. Brain Tissue Preparation

After the behavioral assessment, the rats were sacrificed by intracardial perfusion with saline at 4°C. Then temporal lobes were isolated after clearing blood clots. These tissues were used, respectively, for morphological observation, measurement of Ca^2+^ concentration, Western blot, and TUNEL assay.

### 2.6. Morphological Observation on Mitochondria

As previously described, the sample for morphological observation was prepared [8]. The temporal lobes tissues were fixed with 2.5% buffered glutaraldehyde for 2 hours and then minced into smaller fragments (0.5 mm × 0.5 mm × 1 mm). Next, the tissues were fixed with glutaraldehyde for 1 hour and with 1% OsO_4_ in the same buffer for 1 hour. Then they were dehydrated with alcohol and embedded in araldite. The semithin (300 nm) sections were prepared and then stained with 1% toluidine blue in distilled water at 60°C. Later the sections were trimmed over the neurons to order to gain an overview of the mitochondria of interest under light microscopy. Next, the ultrathin sections (60 nm) were prepared and mounted on nickel grids. The sections were then observed using the Transmission Electron Microscopy JEM-1011 (JEOL, Japan), after being stained with lead citrate and uranyl acetate.

### 2.7. Mitochondria Isolation and Ca^2+^ Measurement

Mitochondria and cytoplasmic fractions were isolated from the temporal lobes using the Mitochondria Isolation Kit for Tissue (Beyotime, China). Next, the Ca^2+^ in cytoplasm and mitochondria were, respectively, detected by the Cytoplasmic Ca^2+^ Concentration Quantitative Determination Kit (Genmed, USA) and the Mitochondrial Ca^2+^ Concentration Quantitative Determination Kit (Genmed, USA). Then the fluorescence microplate reader was used to quantify the Ca^2+^ concentration.

### 2.8. Measurement of Mitochondrial Membrane Potential

Reduction in mitochondrial membrane potential (Δ*ψ*m) is an indicator for mitochondrial and cellular dysfunction [9]. The Δ*ψ*m was detected using JC-1 Mitochondrial Membrane Potential Assay Kit (Beyotime, China). The intensities of red (excitation/emission wave length = 525/590 nm) fluorescence and green (excitation/emission wave length = 490/530 nm) were quantified by the Olympus IX51 inverted microscope system (Olympus, Japan). Then the Δ*ψ*m was indicated by a decrease in the red/green fluorescence intensity ratio.

### 2.9. Western Blotting Analysis

A release of cyt c and second mitochondria-derived activator of caspases (Smac) from injured mitochondria into the cytosol and an increase in cleaved caspase-3 indicate cellular apoptosis. The increase of bcl-2, the apoptosis inhibitor, suggests the protection against apoptosis [6, 11, 12]. Therefore, we measured the expression of cyt c, Smac, cleaved caspase-3, and bcl-2 to determine the effect of treatments on cellular apoptosis.

The brain tissues without mitochondria were used to determine the expressions of cyt c and Smac in the cytoplasm while parts of the fresh temporal lobes were used to determine the expressions of bcl-2 and cleaved caspase-3. As previously described [24], the procedure of Western blot was followed. After being homogenized in lysis buffer (Thermo Fisher Scientific, USA), the brain tissues were centrifuged at 14,000 ×g for 15 minutes at 4°C and then the supernatants were collected. Equal amount of protein (30 *μ*g per lane) was resolved by 2x sodium dodecyl sulfate (SDS) with heating and electrophoresis and then transferred onto the nitrocellulose membrane sheets. After being blocked with 5% nonfat milk for 90 minutes at room temperature, the sheets were incubated overnight at 4°C with primary antibodies against cyt c (1 : 1000, Abcam, UK), cleaved caspase-3 (1 : 500, Cell Signaling Technology, USA), bcl-2 (1 : 1000, Santa Cruz Biotech, USA), Smac (1 : 1000, Cell Signaling Technology, USA), and the loading control GAPDH (1 : 5000, Abcam, UK). Then the membranes were washed with Tween 20 in Tris buffered saline (TTBS, 100 mM Tris–HCl, pH 7.5, 0.9% NaCl, 0.1% Tween 20). After being incubated with secondary antibodies and washed again, the immunoreactive polypeptide bands were visualized by the Enhanced Chemiluminescence Western blot Detection Reagents (Thermo Fisher Scientific, USA) and their densities were quantified by the Automatic Chemiluminescence Imaging Analysis System (Tanon-5200, Tanon, China). The changes of protein expression were calculated as target protein expression/GAPDH expression ratio.

### 2.10. TUNEL Staining

A part of temporal lobes was sliced after being fixed with 4% paraformaldehyde and then it was dehydrated by saccharose phosphate-buffered saline (PBS). TUNEL assay was conducted using the In Situ Cell Death Detection Kit (Roche, USA). The slices were counterstained by 4′,6-diamidino-2-phenylindole (DAPI, Beyotime, China) and covered by Antifade Mounting Medium (Beyotime, China). Fluorescence microscopy images were observed by the Olympus IX51 inverted microscope system (Olympus, Japan). Apoptosis index (AI) was defined as the mean percentage of TUNEL-positive cells out of six cortical microscopic fields (×400 magnification). The relevant personnel were blind to treatment assignments.

### 2.11. Statistical Analysis

The continuous data was presented as mean ± SD and one-way ANOVA followed by Bonferroni correction test was used to analyze the group difference. In terms of the neurobehavioral impairment, the ordinal outcome was transferred to a dichotomous outcome, effective rate, by dividing the SAH rats into two groups, including those with no or slight impairments and those with moderate or severe impairments and then Chi-square test or Fisher exact test was applied to analyze the group difference. A *P* value of less than 0.05 was considered statistical significance. The statistical analysis was performed using IBM SPSS Statistics 24.0 and the figures were drawn using GraphPad Prism software 6.0.

## 3. Results

### 3.1. Neurobehavioral Impairment

**Figure 1:**
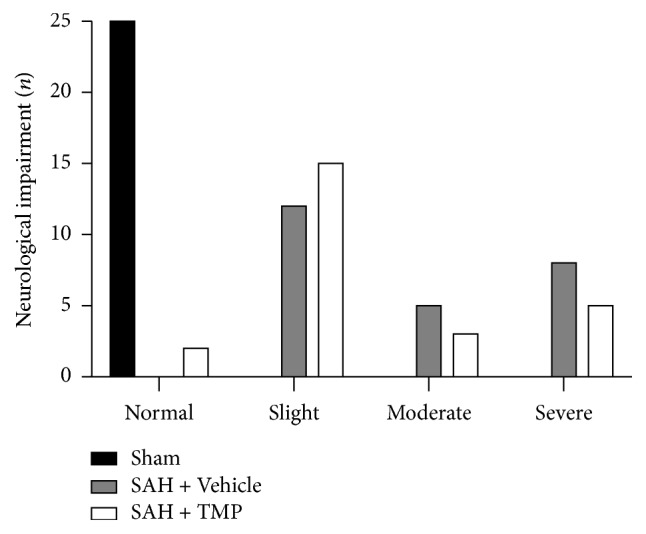
Neurological impairment numbers of animals after SAH. There was no statistical difference between the SAH + TMP group and SAH + Vehicle group (*χ*^*2*^ = 0.936, *P* = 0.52) (*n* = 25 in each group) although there was a trend that TMP resulted in less neurological impairment.

None of the rats died or suffered from neurobehavioral impairments in the sham-operation group as well as blood clots around basilar arteries and temporal lobes (Figure 1). There was no statistical difference of the effective rate between the SAH group treated with TMP and that with saline (*χ*^*2*^ = 0.936, *P* = 0.52) although there was a trend that TMP resulted in less neurological impairment.

### 3.2. Morphological Observations for Mitochondria

**Figure 2:**
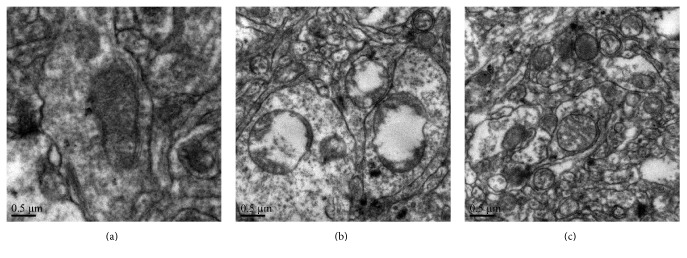
Electron photomicrographs of mitochondria after SAH and protective effects of TMP. (a) A mitochondrion with normal shape from the sham-operation group. (b) Swelling mitochondria with collapsed cristae, disruptive membranes from the SAH + Vehicle group. (c) Mitochondria with much better shape from the SAH + TMP group. Scale bar indicates 0.5 *μ*m.

The transmission electron microscopy found normal shape, intact membrane and cristae, and dense matrix space for the mitochondria in the sham-operation group (Figure 2(a)). In the SAH group treated with saline, the mitochondria swelled irregularly, mitochondrial vacuoles appeared, and the membrane and cristae fractured and became fuzzy (Figure 2(b)). In the SAH group treated with TMP, the injuries of mitochondria such as swelling and fracture appeared milder than the group treated with SAH (Figure 2(c)).

### 3.3. Cytoplasmic and Mitochondrial Ca^2+^ Concentrations

Ca^2+^ concentration in both cytoplasm and mitochondria after experimental SAH greatly increased. Compared to the SAH group treated with saline, lower concentration of Ca^2+^ in cytoplasm and mitochondria was found in the group with TMP and these differences were statistically significant (Table 1).

### 3.4. Mitochondrial Membrane Potential

**Figure 3:**
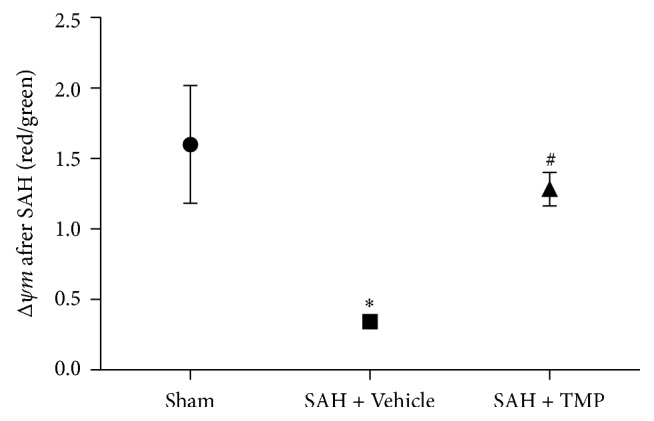
Δ*ψ*m of animals after SAH and protective effects of TMP. Δ*ψ*m decreased dramatically after SAH, which means depolarization of mitochondria. Treatment with TMP greatly alleviated the decrease of Δ*ψ*m. Data represent the mean ± SD of 3 groups (*n* = 5 in each group). (^*∗*^*P* < 0.05 versus sham-operation group; ^#^*P* = 0.515 versus sham-operation group).

Compared to the sham-operation group (1.60 ± 0.42), Δ*ψ*m in the SAH treated with saline group was significantly decreased, indicating mitochondrial depolarization. Δ*ψ*m in the SAH treated with TMP (1.28 ± 0.19) was higher than that in the group with saline (0.34 ± 0.01) and this difference was statistically significant (*P* < 0.05). However, there was no statistical difference of Δ*ψ*m between the sham-operation and the TMP group (*P* = 0.515). (*n* = 5 in each group) (Figure 3).

### 3.5. Expressions of cyt c, Smac, Cleaved Caspase-3, and bcl-2

**Figure 4:**
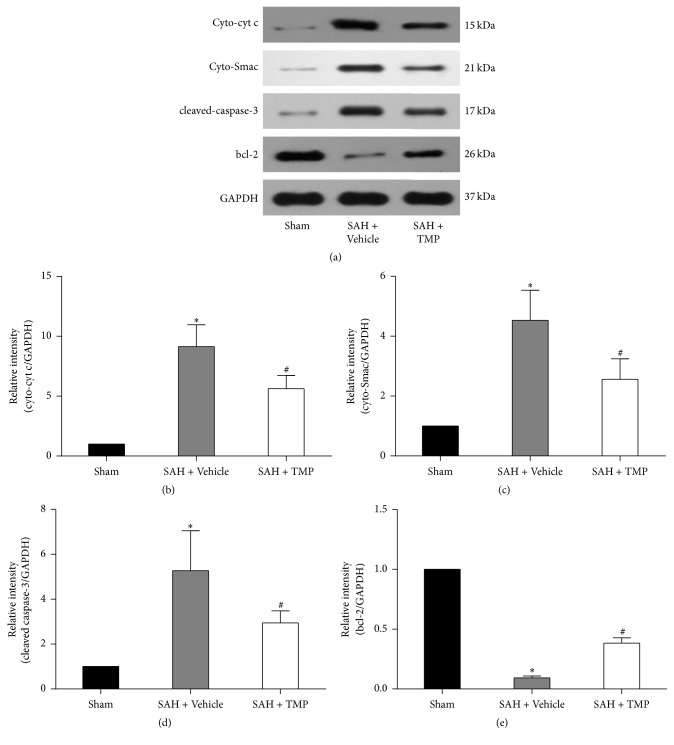
Expressions of cyt c and Smac, cleaved caspase-3, and bcl-2. (a) for Western blotting images; (b) for cyt c in different groups; (c) for Smac; (d) for cleaved caspase-3; (e) for bcl-2. The bar graph shows the ratio of the cyt c, Smac, cleaved caspase-3, and bcl-2 integrated density value (IDV) to GAPDH IDV for each experimental condition. Data represent the mean ± SD of 3 groups (*n* = 5 in each group). As figures showed, the increase of cytoplasmic cyt c and Smac took place after SAH and it was significantly inhibited by TMP treatment. Sham for sham-operation group; SAH for subarachnoid hemorrhage; TMP for tetramethylpyrazine; GAPDH for glyceraldehyde 3-phosphate dehydrogenase; cyt c for cytochrome c; Smac for second mitochondria-derived activator of caspases; bcl-2 for B-cell lymphoma 2. (^*∗*^*P* < 0.05 compared with the sham-operation group, ^#^*P* < 0.05 compared with the SAH + Vehicle group.)

Cyt c and Smac in cytoplasm and cleaved caspase-3 in the SAH with saline group were much higher than those in the sham-operation group. Compared to the SAH group treated with saline, the expressions of these proteins were much lower in the group with TMP and these differences were statistical significant. The expression of bcl-2 was much lower in the SAH with saline group or TMP group than the sham-operation group. However, the differences of cyt c, Smac, cleaved caspase-3, and bcl-2 were statistically significant between the TMP group and the sham-operation group.  Figure 4 detailed the representative images from Western blotting analysis and the statistical results.

### 3.6. TUNEL-Positive Cells

**Figure 5:**
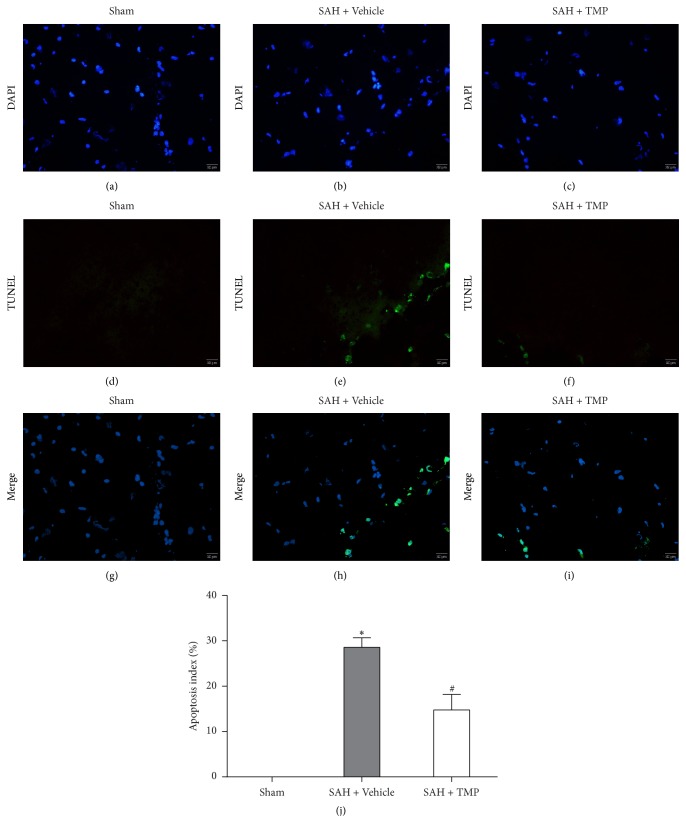
TUNEL-positive and apoptosis index. (a–c) Representative microphotographs of DAPI-positive cells (blue dots); (d–f) representative microphotographs of TUNEL-positive cells (green dots); (g–i) representative microphotographs merging DAPI-positive cells and TUNEL-positive cells. (j) Apoptosis index (AI) was defined as the mean percentage of TUNEL-positive cells out of all DAPI-positive cells in six cortical microscopic fields (×400 magnification). As figures showed, the apoptosis triggered by SAH was improved by TMP. Bars represent the mean ± SD (*n* = 5 in each group) (^*∗*^*P* < 0.05 compared with the sham-operation group, ^#^*P* < 0.05 compared with the SAH+ Vehicle group.) Scale bar indicates 20 *μ*m.

Figures 5(a)–5(i) showed the representative microphotographs of DAPI-positive cells, TUNEL-positive cells and the merging. The apoptotic cells in the SAH with saline group dramatically increased compared to the sham-operation group. The apoptosis index (AI) which was the TUNEL-positive cells/DAPI-positive cells ratio in the SAH with TMP group was much less than that in the group with saline and this difference was statistically significant (*P* < 0.05); However, TMP group resulted in higher AI than the sham group (Figure 5(j)). (*n* = 5 in each group).

## 4. Discussion

Cell apoptosis after SAH is one of the predominant mechanisms in developing EBI [10, 24]. Although the benefit of TMP to cellular apoptosis for cerebrovascular diseases is determined in laboratory experiments [12, 19, 20, 25], only a few are conducted in SAH animal models. Our study demonstrates the protective effect of TMP against cell apoptosis after experimental SAH, which is of significant therapeutic implication. In addition, we investigated the impact of TMP on the potential caspase-3 apoptotic pathway. We found that the acute treatment of TMP could suppress the cyt c, Smac and cleaved caspase-3, and apoptotic activators and elevated bcl-2, the apoptotic inhibitor [26]. Also TMP resulted in slighter mitochondrial impairments, in terms of morphology, calcium overloading, and Δ*ψ*m. All these findings suggest that the protective effect of TMP against cell apoptosis after SAH is probably associated with mitochondrial-dependent caspase-3 apoptotic pathway, which provides additional preclinical evidence of TMP for SAH. However, the treatment TMP did not show significant superiority to saline in terms of neurobehavioral functioning 24 hours after SAH though it potentially resulted in better performance. Considering the dose-dependent effect of TMP for ischemia [12, 22], further studies investigating TMP in higher dosage are needed.

A previous study revealed that pretreatment of TMP reduced caspase-3 in the SAH model [20]. However, prevention from EBI is inconsistent with clinical practice as SAH often attacks patients with aneurysm suddenly [1]. Instead, we treated SAH with TMP 15 minutes after establishing the SAH model. Therefore, the preclinical evidence generated from our study is more probably to be transferred to the clinical.

Mitochondria are considered one of the major organelles involved in cell apoptosis [27]. Ca^2+^ overloading in mitochondria and the subsequent depolarization of Δ*ψ*m are the key indicators of mitochondrial injury [6]. A considerable amount of evidence found rapid Ca^2+^ influx right after the aneurysm rupture and Ca^2+^ overloading was at the very early stage of the mitochondrial injury [6]. Therefore, blocking Ca^2+^ overloading may avoid initiating the apoptotic cascade and further safeguard the patients from EBI [3, 28]. In addition, the striking finding that TMP almost reversed the mitochondrial depolarization suggested that TMP might be a mitochondrial calcium channel blocker. It is recently found that the Ca^2+^ uptake into mitochondria is mediated by mitochondrial calcium uniporter (MCU), a unique ion channel with high conductance and selectivity [29]. Our previous study has demonstrated that the blockage of MCU could reduce intracellular calcium overloading and thus alleviate EBI after SAH [30]. Taken together, TMP is probably a MUC blocker, which has never been reported though it has been regarded as a calcium antagonist in smooth muscle cell [31]. To further examine this possibility, other studies such as observations of the structural changes of the MCU and experimental use of MCU openers (e.g., polyamine like spermine a) following the treatment of TMP are needed.

Caspase-3 is located at the final of apoptotic pathway [32]. It is often used as a representative activator for apoptosis. It would be more valuable to determine whether TMP could reverse the upstream impairment of caspase-3 level. Smac is a major determinant in the mitochondria-dependent apoptotic pathway. It can drive or amplify the release of cyt c and following cyt c further elevate the production of caspase-3 [33, 34]. Our study found TMP could suppress cyt c, Smac, and caspase-3, which implied that TMP could also ameliorate the earlier commencement of the apoptosis. But the releasing of mitochondrial Smac is often suppressed by bcl-2 [26]. Our study also found the enhancement of bcl-2 after the treatment of TMP, which suggested the global impact of TMP on the apoptotic pathway.

In addition to initiating cellular apoptosis, impaired mitochondria may also lead to cellular necrosis due to the depletion and insufficient generation of ATP and serious influence on the normal cellular communication through Ca^2+^ signals, particularly in the central nervous system [7, 35, 36]. Therefore TMP may also reverse cellular necrosis. Other potential benefits of TMP may include reducing the overproduction of reactive oxygen species, ameliorating cellular inflammatory response, and maintaining vasorelaxation by enhancing eNOS [19, 37, 38].

In clinical settings, nimodipine is the only drug approved for SAH; however, its effectiveness on neurological performance is unsatisfactory although a series of high-quality evidences indicate its therapeutic potential in vasospasm [39, 40]. Clinical studies have suggested the dissociation between vasospasm and neurological functions [41]. According to our best knowledge, up to now there is no successful neuroprotective drug for SAH [42]. TMP could be a promising new drug candidate for SAH.

Limitations include the uncertain identity of the apoptotic cell and mitochondria. Further evaluation on antibodies against neuronal marker neuronal nuclear protein (NeuN) and astrocyte marker glial fibrillary acidic protein (GFAP) following the analysis of the TUNEL-positive cells is needed [43]. Cellular identity of mitochondria is also required as the mitochondria in our research may come from neurons or astrocytes.

## 5. Conclusions

TMP could suppress the mitochondrial apoptosis-relative pathway after SAH. These results suggest that TMP may be a new neuroprotectant for the treatment of SAH.

## Figures and Tables

**Table 1:** Ca^2+^ concentration in the cytoplasm and mitochondria.

	Cytoplasmic Ca^2+^ (10^−2^ nmol/*μ*g)	Mitochondrial Ca^2+^ (10^−2^ nmol/*μ*g)
Sham-operation group	24.03 ± 8.10	78.69 ± 6.46
SAH + Vehicle group	237.94 ± 15.13^*∗*^	281.47 ± 19.82^*∗*^
SAH + TMP group	81.95 ± 11.26^#^	179.96 ± 20.11^#^

*Note*. Data represent the means ± SD of 3 groups (*n* = 5 in each group) (^*∗*^*P* < 0.05 versus sham-operation group; ^#^*P* < 0.05 versus SAH group with vehicle).
